# Fabrication of Ternary Nanoparticles for Catalytic Ozonation to Treat Parabens: Mechanisms, Efficiency, and Effects on *Ceratophyllum demersum* L. and Eker Leiomyoma Tumor-3 Cells

**DOI:** 10.3390/nano12203573

**Published:** 2022-10-12

**Authors:** Apiladda Pattanateeradetch, Chainarong Sakulthaew, Athaphon Angkaew, Samak Sutjarit, Thapanee Poompoung, Yao-Tung Lin, Clifford E. Harris, Steve Comfort, Chanat Chokejaroenrat

**Affiliations:** 1 Department of Environmental Technology and Management, Faculty of Environment, Kasetsart University, Bangkok 10900, Thailand; 2 Department of Veterinary Technology, Faculty of Veterinary Technology, Kasetsart University, Bangkok 10900, Thailand; 3 Department of Soil & Environmental Sciences, National Chung Hsing University, Taichung 402, Taiwan; 4 Department of Chemistry and Biochemistry, Albion College, Albion, MI 49224, USA; 5 School of Natural Resources, University of Nebraska-Lincoln, Lincoln, NE 68583, USA

**Keywords:** advanced oxidation process, catalytic ozonation mechanism, heterogeneous catalysis, paraben degradation, reactive oxygen species, ternary nanocomposites

## Abstract

The use of parabens in personal care products can result in their leakage into water bodies, especially in public swimming pools with insufficient water treatment. We found that ferrite-based nanomaterials could catalytically enhance ozone efficiency through the production of reactive oxygen species. Our objective was to develop a catalytic ozonation system using ternary nanocomposites that could minimize the ozone supply while ensuring the treated water was acceptable for disposal into the environment. A ternary CuFe_2_O_4_/CuO/Fe_2_O_3_ nanocomposite (CF) delivered excellent degradation performance in catalytic ozonation systems for butylparaben (BP). By calcining with melamine, we obtained the CF/g-C_3_N_4_ (CFM) nanocomposite, which had excellent magnetic separation properties with slightly lower degradation efficiency than CF, due to possible self-agglomeration that reduced its electron capture ability. The presence of other constituent ions in synthetic wastewater and actual discharge water resulted in varying degradation rates due to the formation of secondary active radicals. ^1^O_2_ and ^•^O_2_^−^ were the main dominant reactive species for BP degradation, which originated from the O_3_ adsorption that occurs on the CF≡Cu^(I)^–OH and CF≡Fe^(III)^–OH surface, and from the reaction with ^•^OH from indirect ozonation. Up to 50% of O_3_-treated water resulted in >80% ELT3 cell viability, the presence of well-adhered cells, and no effect on the young tip of *Ceratophyllum demersum* L. Overall, our results demonstrated that both materials could be potential catalysts for ozonation because of their excellent degrading performance and, consequently, their non-toxic by-products.

## 1. Introduction

Parabens have been utilized widely as antimicrobial preservatives in many cosmetic and body care products for several decades. Swimming pools are a major sink of parabens from human activity, followed by municipal domestic sewage [1]. Although the use of chlorination and salinity in pool water should eliminate paraben residuals, Terasaki and Makino [2] found that parabens were, in turn, active ingredients for disinfection by-products (that is, chlorinated parabens). Given that human activity is a significant source of parabens, it is not surprising that urban areas are experiencing an increase in paraben-contaminated water. Up to 228 ng L^−1^ of parabens was detected in 39 different kinds of swimming pools from various places in China, such as hotel, residential, and commercial pools [3]. The improper discharge of pool water without any pretreatments has been reported and could consequently cause paraben contamination to any nearby natural receiving water and eventually to drinking water [4]. Some parabens were detected in 58 fish samples from 20 species collected from a fish market near Manila Bay, the Philippines [5]. While all parabens are categorized as endocrine disruption chemicals, butylparaben is more prone to induce vitellogenin production in male fish [6], and it has been reported to have higher estrogenic activity than other parabens [7]. This evidence confirms that a conventional water treatment system is insufficient; consequently, the development of vigorous treatment technology that results in non-toxic byproducts is needed.

Ozonation is an effective oxidant for rapidly transforming refractory pollutants due to its high oxidation potential (2.07 V). Despite such a high efficiency, using ozone alone (O_3_) can be overcome by other oxidation processes due to its oxidant solubility in water and the high energy requirement to provide sufficient O_3_ during utilization. Catalytic ozonation via a heterogeneous route has been used widely because it allows the recovery and reuse of materials, as well as the ability to enhance the mass transfer efficiency for treating target contaminants. During catalytic ozonation, utilization of O_3_ was reduced compared to the use of O_3_ alone [8]. The mechanisms of direct ozonation and indirect ozonation via the formation of ^•^OH have been well-established, but to a lesser extent than those of heterogeneous catalytic ozonation, especially with multiplex nanocomposites. This calls for these particular nanocomposites, which can strongly facilitate active radical generation (^•^OH, O_2_^•−^, ^1^O_2_), to be fabricated by simple co-precipitation methods.

Several nanocomposites, such as transition metal ferrites (MFe_2_O_4_; M = Zn, Mn, Co, Ni, and Cu) and iron oxides (Fe_x_O_y_), have been shown to deliver outstanding performance in enhancing oxidation processes via electron transfer [9]. These materials have gained attention due to their natural abundance, low-cost fabrication methods, and chemical stability. The spinel ferrites exhibit crystalline stability and can support recyclability because they can provide magnetic separation properties and high catalytic activity [10]. Although Fe_x_O_y_ is notable for providing higher magnetism, its activation ability towards PS activation is still unsatisfactory [11]. Yan et al. [12] found that catalytic O_3_ with α-Fe_2_O_3_ resulted in slower generation rates of active species compared to α-FeOOH and Fe_3_O_4_ due to the lack of surface Lewis acid sites on α-Fe_2_O_3_ that prevent the interaction of adsorbed O_3_ on the surface of Fe^(III)^. Nonetheless, an Fe-based catalyst can serve as a suitable catalyst for O_3_ in degrading polychlorinated dibenzo-p-dioxins and polychlorinated dibenzofurans at low temperatures, providing that the catalyst has a high surface area of up to 300 m^2^ g^−1^ [13]. Several researchers have highlighted the benefit of using a multiplex catalyst in oxidation processes that can empower electron mobility by adding more than one element. For example, molybdenum oxide was used as a catalyst promoter in the CuO/Al_2_O_3_ synthesis process, resulting in the formation of Cu-Mo-Al complexes to enhance the ozonation ability of dimethyl sulfide [14]. Li et al. [15] replaced active sites of iron oxides that had a weaker catalytic activity with other metal ions with various valence states, such as Mn, to become a magnetic Fe_3_O_4_-MnO_3_ nanocomposite to enhance the activation of peroxymonosulfate. Complex nanocomposites of bimetal oxides deposited on Zn-Fe silicate were found to trigger chain reactions of O_3_ to form more hydroxyl radicals during catalytic ozonation [16].

While these complexes are not commonly used in a conventional water treatment system, this can be altered by introducing the right dopants into the nanocomposites and utilizing their unique properties. The construction of nanocomposites with a metal-free catalytic material, such as graphitic carbon nitride (g-C_3_N_4_), is also an interesting choice for providing superior magnetic separation properties [17]; however, its catalytic ozonation performance, especially with parabens, remains unknown. Given that several synthesis methods are available, co-precipitation is a very convenient approach and does not require any toxic solvent during synthesis, which is also feasible for large-scale production.

In the current study, several transition metals were selectively investigated in the search for better-mixed transition metal oxides for higher catalytic ozonation with a strong magnetic separation properties. The physicochemical characteristics of the material before and after ozonation were compared side-by-side to explain their mechanistic behavior. To ensure the system applicability, the effects from different water matrices and other environmental variables, which would have an impact on the paraben degradation rates, were also investigated. The reaction mechanism was determined through scavenging experiments using various chemical probes and changes in element composition using X-ray photoelectron spectroscopy (XPS). The transformation of the selected parabens during degradation was explained in detail. The toxicity of O_3_-treated was evaluated water on *Ceratophyllum demersum* L. As parabens have been known to possess endocrine-disruptive properties, we also evaluated their impact on Eker Leiomyoma Tumor-3 cells following exposure to the different proportions of O_3_-treated water. 

## 2. Materials and Methods

Chemical vendors and manufacturers, analytical procedures, catalyst characteristics, and preparation of ELT3 cell culture are detailed in the Supplementary Materials (Sections S1–S4).

### 2.1. Catalyst Synthesis

All metal ferrite nanocomposites (MF) were synthesized through a co-precipitation method followed by calcination, as modified from our previous study [17]. A mixture of 0.2 M MSO_4_ (CuSO_4_, NiCl_2_·6H_2_O, CoCl_2_·6H_2_O, MnSO_4_·H_2_O), 0.4 M FeCl_3_·6H_2_O, and distilled water (DI) at a ratio of 1:1:2 (*v*/*v*) was heated under a constant stirring at 80 °C for 1 h. A heated solution of 8 M NaOH was used to slowly adjust the pH to 10.5 within 1 h and then the mixture was continuously stirred for 1 h. To obtain uniform particles, the mixture at room temperature was washed with DI water until a neutral pH was reached. The remaining particles were washed twice with ethanol prior to drying overnight in a hot-air oven at 95 °C. The final CuFe_2_O_4_/CuO/Fe_2_O_3_ nanocomposites (CF) were calcined at 550 °C for 3 h. For CF/g-C_3_N_4_ synthesis (CFM), melamine was mixed with the oven-dried particles at a ratio of 2:1 (*w*/*w*) before calcining at 550 °C. All materials were kept in a desiccator until use.

### 2.2. Typical Experimental Setup 

All experiments were performed in a 250 mL beaker. Mixed parabens, consisting of methylparaben (MP), ethylparaben (EP), propylparaben (PP), and butylparaben (BP), at a concentration of 10 µM each were prepared in DI water and used altogether in one batch. As BP is frequently found in natural receiving water, BP degradation was selectively presented, unless stated otherwise. The as-obtained nanocomposites (CF or CFM) of 20 mg were added into the 100 mL mixed parabens solution, equivalent to 0.2 g L^−1^. Before purging with O_3_, each solution was stirred for 30 min to ensure no adsorption had occurred on the material surface. An ozone flux of 10 mg min^−1^ was continuously bubbled along with magnetic stirring. Samples were taken periodically at a designated time within 40 min, involving filtering with a 0.45 µm PTFE syringe filter before transferring to vials for further paraben analysis.

### 2.3. Operational and Influential Effect Experiments

The influential effect experiments were performed using the above protocol while each effect was individually varied. We selected chlorine concentration (1–4 mg L^−1^), hardness (90.28–361.10 mg L^−1^ as CaCO_3_), and bicarbonate (HCO_3_^−^; 100–140 mg L^−1^) as variables that may be present in discharge water. Trichloroisocyanuric acid (TCCA) was used for chlorine variation content as it is the most-selected type of disinfectant for swimming pools and can provide up to 90% available chlorine in the form of hypochlorite (OCl^−^).

Because real discharge pool water is usually mixed with naturally occurring other constituents, including a high loading of organic matter and several kinds of personal care products, the effect of water matrices was determine in four different kinds of water matrices (DC: discharge chlorine pool water, DS: discharge saline pool water, NW: natural receiving water, and MW: municipal wastewater). The DC and DS were collected from a public swimming pool located in Kasetsart University, Bangkok, Thailand. The NW was collected from a natural water basin (13.854640, 100.570256) that received treated pool water, while the MW was collected from an overcrowded residential area located in Chatujak district, Bangkok, Thailand. The basic water qualities were shown in the Supplementary Materials (Table S1). Unlike typical experiments, the BP was selected as the main target to represent all parabens; therefore, the initial concentration was raised by 5-fold.

### 2.4. Scavenging Experiments

To evaluate the possible degradation mechanism of BP in the catalytic ozonation system, we spiked a radical scavenger—tertbutyl alcohol (TBA), p- benzoquinone (pBQ), furfuryl alcohol (FFA), or dimethyl sulfoxide (DMSO)—in each experimental unit and maintained the final concentration ratio of BP to these scavengers at 1:5000, 1:500, 1:500, and 1:5000, respectively. These scavengers served as sources of the hydroxyl radical (^•^OH), superoxide radical (O_2_^•−^), singlet oxygen (^1^O_2_), and electrons (e^−^).

### 2.5. Recyclability Testing

Both CF and CFM were evaluated for their recyclability following the catalytic ozonation process achieved from the five-cycle experiments. The typical experiments were followed as discussed in Section 2.2, but the BP was selected as the main target contaminant. Freshly contaminated water was introduced into the O_3_ reactor for up to 8 h. Sampling was made every 30–60 min for residual BP in the solution. Catalysts were reused after manual (CF) or magnetic (CFM) separation from the solution using a neodymium magnet. After the fifth cycle, samples were oven-dried at 105 °C overnight prior to further crystallinity and surface analysis using FTIR, XRD, and XPS. The loss of material during each cycle was also monitored.

### 2.6. Toxicological Assays 

*Ceratophyllum demersum* L. plants were purchased from a local market in Bangkok, Thailand. The top portion (6–13 cm) of each plant was selected with a dry weight of approximately 2.7–2.9 g. Each portion was grown in an 800 mL PVC bowl filled up with 500 mL of effluent water, which was O_3_-treated water at various proportions (0–100%). This water was mixed with water and used in matrices for growing the selected *Ceratophyllum demersum* L. The plants were collected for physical evaluation after 5 days of exposure to the effluents. 

As parabens can interfere with the reproductive hormones, we used an in vitro bioassay based on the Eker Leiomyoma Tumor-3/Eker uterine leiomyoma cell line (ELT3) purchased from ATCC (Manassas, VA, USA). This cytotoxicity evaluation is rapid and reliable, and most importantly, negates the need to conduct whole-organism-level tests (Reference). Cell viability tests were performed for O_3_-treated water at varying amounts with DI water to ensure that there was no induced cytotoxicity in these cells. Details of culture methods are provided in Supplementary Materials (Section S4).

## 3. Results and Discussion

### 3.1. Nanocomposite Selection

**Figure 1:**
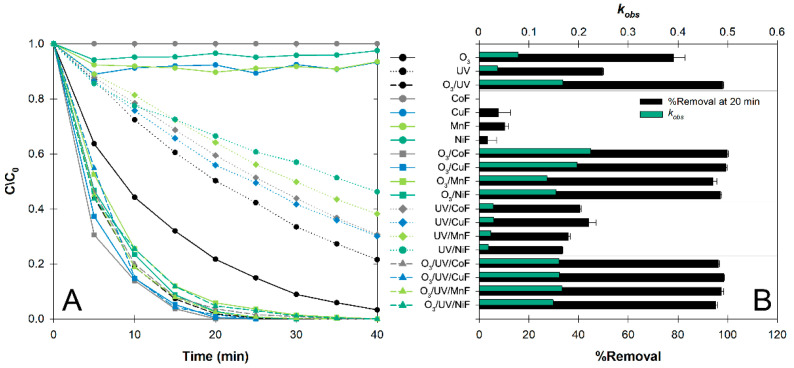
Degradation of butylparaben (BP) under different treatment systems: (**A**) degradation kinetics, and (**B**) observed rate constant (*k_obs_*) and %removal at 20 min (Catalyst: 0.2 g L^−1^, [BP_o_]: 10 µM, pH 7, 25 °C). Error bars indicate ± standard deviation.

Various types of treatments [(1) individual oxidant (O_3_, UV); (2) synthesized nanocomposite alone (MF; M^(II)^ = Co^(II)^, Cu^(II)^, Mn^(II)^, and Ni^(II)^); (3) O_3_/MF; (4) UV/MF; and (5) O_3_/UV/MF] were tested to compare the observed pseudo first-order rate constant (*k_obs_*) and %removal at 20 min (BP in Figure 1; MP, EP, and PP in Figures S1–S3). Overall, BP degradation efficiency was in the order of O_3_/MF > O_3_/UV/MF > O_3_ alone > UV alone > UV/MF > MF alone. Ninety-five percent removal of BP was observed within 40 min using O_3_ alone, while only up to 8% removal was observed when MnF was used, even in the dark, indicating that only slight adsorption occurred on the material surface (Figure 1A). Although the UV-assisted O_3_ and O_3_/UV/MF showed slightly better performance than the others, the enhancement of catalytic ozonation from the UV was unlikely. Some transition metal ferrite nanocomposites used in this study were identified as having a narrow bandgap, which is not an appropriate energy band for UV light. Aside from the UV radiation that only accounts for less than 5% of the solar spectrum, using any light may suffer from the catalysts or any suspended solids that can increase impeding light penetration. For example, under our UV/MF condition, the BP removal was reduced by 60%, and the *k_obs_* was decreased by 3-fold (Figure 1B).

Although CoF/O_3_ resulted in faster degradation performance than CuF/O_3_ in all parabens, the use of CoF resulted in evidence of agglomeration during utilization, which would easily hinder magnetic separation and may further obstruct the reusability of the material. Morphologically speaking, the spinel CuFe_2_O_4_ is more structurally stable and should provide a better magnetic response after simple modification than the CoFe_2_O_4_ [18]. The outstanding attribute of CuFe_2_O_4_ is its smaller pore volume expansion and its ability to donate more electrons, making it favorable for electron transfer [19]. Therefore, CuF was selected as the more suitable catalyst for enhancing the ozonation process.

### 3.2. CF Nanocomposite Properties

#### 3.2.1. Physical and Chemical Characteristics 

**Figure 2:**
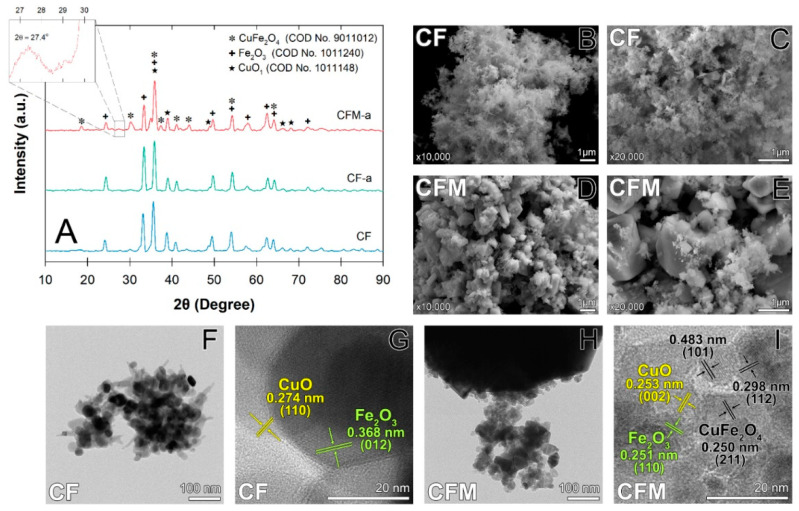
CuFe_2_O_4_/CuO/Fe_2_O_3_ ternary nanocomposites: (**A**) XRD patterns of catalysts before and after catalytic ozonation, (**B**,**C**) CF SEM images, (**D**,**E**) CFM SEM images, (**F**,**G**) CF TEM images, (**H**,**I**) CFM TEM images.

From a catalytic activity standpoint, bare CuFe_2_O_4_ could be used as a backbone for mixed transition metal oxides [20]. Here, we characterized the nanocomposites and revealed that it was the ternary catalyst of CuFe_2_O_4_/CuO/Fe_2_O_3_, denoted as CF, which was later decorated with g-C_3_N_4_, denoted as CFM, while CF-a and CFM-a represented the used material in the O_3_-system to remove parabens. Using the crystallography open database (COD; No 9011012), diffraction peaks of 18.58°, 30.23°, 35.87°, 37.38°, 41.10°, 44.07°, 54.29°, and 64.21°, which are ascribed to the (101), (112), (211), (311), (222), (113), (400), (422), and (440) lattice planes of CuFe_2_O_4_, are more intensified with CFM than with the CF (Figure 2A). This finding indicated that the recrystallization was completed during the calcination processes. The small peaks observed at 35.87°, 38.98°, 48.96°, and 68.25°, corresponding to the reflection of the (110), (111), (202), and (113) planes of CuO, showed that both materials (CF-a versus CFM-a) lost CuO content after O_3_ treatment, even after undergoing the thermal process [21]. The hematite phase is indicated by the diffraction peaks of 33.40° and 35.6°, which are ascribed to the (104) and (110) planes on both materials. These peaks confirmed that as-obtained CF and CFM can be successfully fabricated using co-precipitation methods.

It appeared that pure-g-C_3_N_4_ had diffraction peaks of 27.4°, which also weakly appeared in the CFM nanocomposites, indicating that the calcination process may have restructured the material (Figure 2A). While Wang et al. [22] showed that losing g-C_3_N_4_ after calcination was possible, we believe that the calcination process causes the dispersion of CuFe_2_O_4_ particles and may overshadow the diffraction peaks of g-C_3_N_4_. Overall, the XRD diffraction peaks of CF-a showed slightly higher intensities than for CFM-a, indicating that ozonation may reduce material crystallinity. With a higher O_3_ flux, recyclability of CFM may be affected, and therefore using CF would probably be more tangible for practical operation.

The morphological features of CF showed its fluffy texture consisting of nanoparticles (15–60 nm), which are interconnected among the three transition metal oxides (Figure 2B,C). High-magnification of the CF TEM images revealed a mixture of the platelet-like hematite particles that are tightly attached to the spherical shape of CuFe_2_O_4_ and CuO (Figure 2F,G). Unlike CF, the CFM nanoparticles tended to agglomerate, resulting from recrystallization during the calcination stage with melamine. The majority of CFM particles were irregular platelets, with surrounding smaller cubes and spheres with an average size of 20.84 nm, which corresponded with the average size obtained from the XRD results (Figure 2D,E, Tables S2 and S3). In addition, CFM-a had a higher remaining content of CuFe_2_O_4_ than CF-a due to its agglomeration, as g-C_3_N_4_ can serve as a particle substrate. While both N_2_ adsorption/desorption isotherms confirmed their uniformly mesoporous material, changes following calcination were observed for the pore size (~37.5 to 32.7 nm) and the BET surface area (24.13 to 2.91 m^2^ g^−1^), indicating that there might be some compaction and distortion of the crystal structures from the use of the g-C_3_N_4_ substrate (Figure S4).

The measured lattice fringes of 0.250, 0.298, and 0.483 nm from the CFM HRTEM image corresponded to the CuFe_2_O_4_ planes (Figure 2I). In addition, these lattice spacings of CuO and Fe_2_O_3_ of 0.253 nm and 0.251 nm, which are consistent with the (002) and (110) plane of XRD results, also revealed that g-C_3_N_4_ and CF nanoparticles were unified with structural densification and uniform distribution (Figure 2G,I). The lack of the CuFe_2_O_4_ lattice plane in the CF HRTEM image was also in accordance with the small portion of CuFe_2_O_4_ revealed from its XRD results (Figure 2A), signifying the absence of CuFe_2_O_4_ during the CF synthesis and confirming that g-C_3_N_4_ enabled the magnetism of material via CuFe_2_O_4_ deposition (Figure 2G).

**Figure 3:**
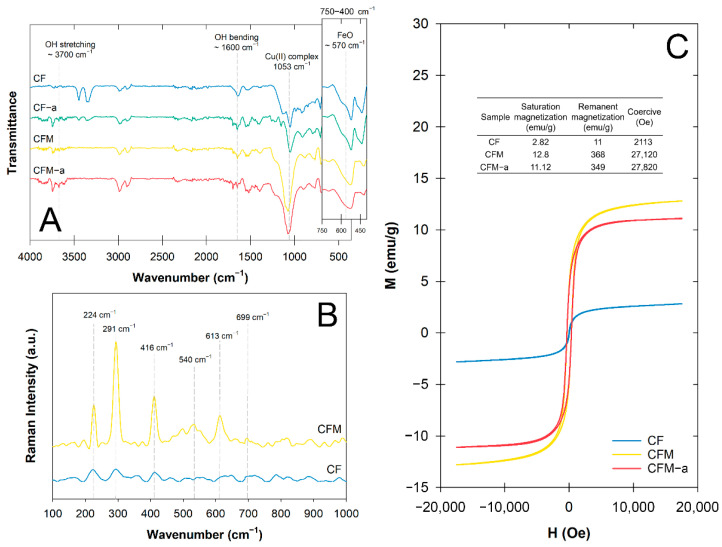
CuFe_2_O_4_/CuO/Fe_2_O_3_ ternary nanocomposites: (**A**) FTIR spectra before and after catalytic ozonation, (**B**) Raman spectra, and (**C**) vibrating sample magnetometer hysteresis loops before and after catalytic ozonation.

The formation of CuO on each nanoparticle was confirmed by the stretching vibration of the Cu-O bands on the FTIR spectra at 437.84, 516.92, and 590.22 cm^−1^ [23] (Figure 3A). The intensities of the non-magnetic CuO peaks showed that the surface functional groups were unchanged after use. By comparing the Raman spectra of the CF and CFM nanoparticles, CFM had six characteristics bands of 224, 291, 416, 540, 613, and 699 cm^−1^ that were well-defined as cubic CuFe_2_O_4_ nanoparticles [24], while those of CF showed a much weaker response (Figure 3B). This finding corresponded to our earlier discussion on the reappearance of CuFe_2_O_4_ in the CFM crystallization (Figure 2A and Figure 3A). Furthermore, some of the CF Raman bands were quite broad with a much weaker band than those of CFM, confirming it was composed of ternary nanocomposites.

All the CF and CFM samples displayed narrow hysteresis loops with the specific saturation magnetization (M_s_) value of CFM (12.8 emu·g^−1^) being 4.5 times higher than that of CF (2.8 emu·g^−1^) (Figure 3C). This could be associated with the presence of CuFe_2_O_4_ in the nanocomposites in the XRD pattern at 18.58°, 37.38°, and 44.07°, and a stronger band at 570 cm^−1^ attributed to the Fe-O for CFM (Figure 2A). Here, the g-C_3_N_4_ increased the inter-particle interaction of these ternary catalyst particles enabling its adherence between microspheres [25]. Only slight changes in the magneticity of the used CFM were observed (~1.68 emu·g^−1^), indicating the material had strong magnetization after several uses and remained intact. The overall results revealed that CFM was more efficiently separated from the external magnetic source making it ideal as an environmental catalyst that could prevent secondary pollutants.

#### 3.2.2. Element Compositions of CF Nanocomposites

**Figure 4:**
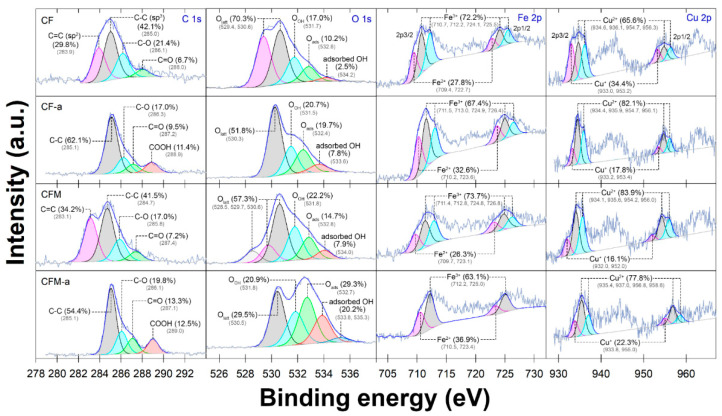
XPS spectra of CuFe_2_O_4_/CuO/Fe_2_O_3_ ternary nanocomposites before (CF, CFM) and after (CF-a, CFM-a) catalytic ozonation (C 1s, O 1s, Fe 2p, and Cu 2p).

The full-survey scan of the XPS spectra showed the existence of C 1s, O 1s, Fe 2p, and Cu 2p, with no evidence of impurities, while the fine scan revealed the elemental valence states and the bonding configuration of these elements (Figure S5 and Figure 4). Five major peaks at binding energies of 283.9 eV, 285.0 eV, 286.1 eV, 288.0 eV, and 288.9 eV in the C1s spectra were assigned to C–C, C–O, C–O–C, C=O, and O=C–O [16]. Additionally, the major peaks at binding energies of 530 eV, 531.7 eV, and 532.8 eV, were attributed to surface lattice oxygen (O_latt_; O^2−^), surface adsorbed oxygen in the hydroxyl group (O_OH_), and the oxygen-containing functional group (O_ads_) (Figure 4). Although only a weak characteristic peak of g-C_3_N_4_ was noticeable in the XRD pattern of CFM-a, we identified no trace of N composition in the N XPS spectra (Figure 2A and Figure 4). We expected that the g-C_3_N_4_ structure might have been deformed to other NO_x_ during the calcination process after facilitating the recrystallization process of the CFM. 

All Fe spectrum displayed dominant doublet peaks located at binding energies of 710.2 eV (Fe 2p_3/2_) and 724.3 eV (Fe 2p_1/2_), which were attributed to Fe^(II)^ and Fe^(III)^ (Figure 4). Similar to the Cu spectrum, two major peaks were observed at binding energies of 934.6 eV (Cu 2p_3/2_) and 954.7 eV (Cu 2p_1/2_), which were attributed to Cu^(II)^, and a small amount of Cu^(I)^ at binding energies of 933.0 eV (Cu 2p_3/2_) and 953.2 eV (Cu 2p_1/2_), which were attributed to Cu^(I)^. Both these results confirmed that the Cu^(II)^ species made up the main proportion in the catalyst. After the deconvolutions of Fe 2p and Cu 2p, the changes in Fe and Cu were clear, indicating that electron transfer had occurred on the catalyst surface following the catalytic ozonation. Notably, there was evidence of the presence of the absorber copper hydroxide (Cu(OH)_2_) at binding energies of 936.1 eV and 956.3 eV, which have arisen from the use of NaOH in the co-precipitation process.

### 3.3. Enhanced Ozone Degradation Performance

**Figure 5:**
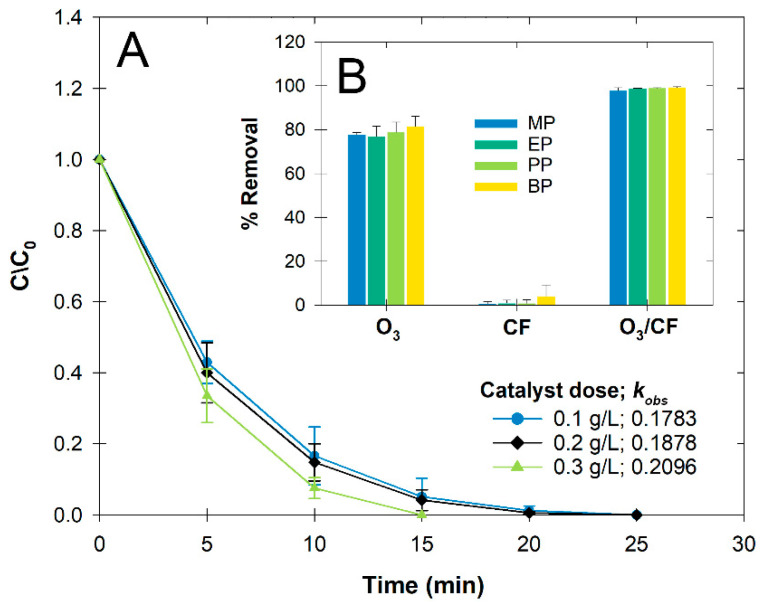
(**A**) Temporal changes of BP at different CF dosages in the O_3_/CF system (Catalyst: 0.2 g L^−1^, [each paraben_o_]: 10 µM, pH 7, 25 °C). (**B**) Paraben removal efficiency at 20 min under different treatments (O_3_, CF, and O_3_/CF), and Error bars indicate ± standard deviation.

The catalytic ozonation performance of the CF and CFM nanoparticles were investigated by the removal of total parabens under varying catalyst doses (0.1–0.3 g L^−1^) (BP in Figure 5; MP, EP, and PP in Figure S6). Because initial CFM tests to enhance ozonation performance revealed insignificantly difference than that of CF in a single batch experiment (data not shown), the CFM was used to compare again in the application study (Section 3.5: Material recyclability). Compared to using O_3_ alone, the O_3_/CF increased the removal efficiency by approximately 20% (inset of Figure 5). Only slight adsorption of BP on the CF surface was observed (<6%). This slight adsorption was expected for ternary inorganic nanocomposites unlike other magnetic porous carbon-based material that were enriched in adsorption sites and were prone to adsorb organic contaminants [26]. Among all parabens tested, we found that BP had higher adsorption activity and faster degradation rates, which could have been due to the length of the ester chains and its possession of more unsaturated C=C bonds that are preferably attacked by the O_3_ molecule [27]. Here, by varying dosage, about 77.2%, 81.1%, and 91.4% of BP were degraded at 10 min of reaction (Figure 5) because the larger specific area created more reactive sites between O_3_ molecules and catalysts, which accelerated the generation of reactive species. Given that the self-scavenging effect has been observed in several studies using higher oxidant doses or excessive amounts of catalyst dosage, we did not observe that phenomenon. This was likely due to the short-lived O_3_ and the beneficial use of the ternary nanocomposites. Therefore, the CF of 0.3 g L^−1^ was selected as the optimum dosage for other successive experiments.

### 3.4. Influential Effects on Degradation Efficiency

**Figure 6:**
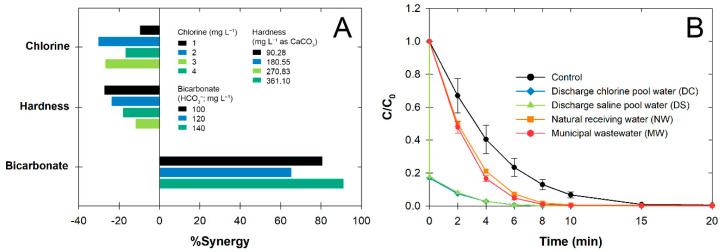
(**A**) Comparison of observed rate constant (*k_obs_*) with different influential effects, (**B**) Temporal changes in BP at different water matrices (catalyst: 0.2 g L^−1^, [BP_o_]: 10 µM, pH 7, 25 °C). Error bars indicate ± standard deviation.

In this part of the study, the BP degradation efficiency following the heterogeneous catalytic oxidation process was tested against the presence of common environmental factors or different water matrices that can only link to the paraben-contaminated water. Given that an alkaline pH can increase ozonation efficiency by several magnitudes from the presence of available OH^−^, we did not select this variable due to its working condition being close to pool water and natural water environmental pH conditions [28]. Following treatment, we reported on the changes in *k_obs_* compared to the control experiment (no effect) (Figure 6). The existence of OCl^−^ caused negative effects on BP degradation by decreasing *k_obs_* by up to 30% due to the presence of secondary chlorine radicals (Figure 6A). Haag and Hoigné [29] found that O_3_ can directly react with OCl^−^ and yield Cl^−^ as a major product (Equation (1)). The generation of reactive chlorine radicals (Cl^•^) and the dichlorine radical (Cl_2_^•−^) may occur during the ozonation in the presence of Cl^−^ with available ^•^OH from the indirect ozonation (Equations (2)–(4)) [30]. Although Yang et al. [31] reported that both chlorine radicals were prone to attacking phenol-containing compounds, the reaction between chlorine radicals and Cl^−^ may be faster than that with parabens. Once the secondary chlorine radicals (Cl_2_^•−^) were formed, they were likely self-scavenged into chloride and BP degradation then ceased (Equations (4) and (5)) [32].

O_3_ + OCl^−^ → 2O_2_ + Cl^−^ (1)

^•^OH + Cl^−^ → ClOH^•−^ (2)

ClOH^•−^ + H^+^ → Cl^•^ + H_2_O (3)

Cl^•^ + Cl^−^ ↔ Cl_2_^•−^ (4)

Cl_2_^•−^ + Cl_2_^•−^ → 2Cl^−^ + Cl_2_ (5)

Hardness is one of the dominant water parameters usually present in natural water. Here, we varied the amount of hardness and found that the presence of hardness negatively affected the catalytic ozonation of BP (Figure 6A). During catalytic ozonation, oxygenated compounds may occur, which initiate the reaction of abundant cations in very hard water, promoting coagulation and possibly suppressing BP degradation [33].

Because TCCA and other water constituents can contribute to the available alkalinity of pool water, the alkalinity is required to be maintained at 60–180 mg L^−1^ as CaCO_3_ [34]. Our results show that the remaining alkalinity could accelerate the BP degradation efficiency using ozonation, which was possibly due to the subsequent increase in pH (Figure 6A). In addition, our CF was prepared in NaOH medium which could also result in the strong intensity of the –OH group on the CF surface (Figure 3A). We believe that this sudden increase in BP degradation mainly resulted from the ^•^OH generation originating from the OH^−^ and O_3_ molecules to generate more of other secondary active radicals, such as O_2_^•−^ and HO_2_ free radicals (HO_2_^•^) (Equations (6)–(9)) [35].

O_3_ + OH^−^ → HO_2_^−^ + O_2_ (6)

HO_2_^−^ + H^+^ → H_2_O_2_ (7)

HO_2_^−^ + O_3_ → O_2_^•−^ + ^•^OH + O_2_ (8)

H_2_O_2_ + O_3_ → HO_2_^•^ + ^•^OH + O_2_ (9)

In real-world applications, more than one type of paraben has been detected in the discharge water, so we used all four parabens as the starting substrate, but the plotted *k_obs_* represents BP only (Figure 6B). The results showed that the paraben degradation rates were higher than the control in all types of water matrices, indicating the presence of several constituents in the water matrices that may have contributed to the generation of secondary active radicals from the specific characteristics of the humic substance surface [36], some of which can facilitate the formation of reactive radicals. Although Haman et al. [37] reported that BP molecules were sensitive to residual chlorines, sudden decreases in BP concentration were possible. However, this was unlikely to occur, because chlorine residuals are susceptible to self-deterioration from maturation basins. The K_oc_ of PP and BP was larger than those of MP and EP by two-fold, suggesting that both PP and BP could be expected to sorb onto suspended solids and the sediment surface [38]. Therefore, we believe that the decrease was also likely a result of the adsorption effect that occurred simultaneously.

### 3.5. Material Recyclability

**Figure 7:**
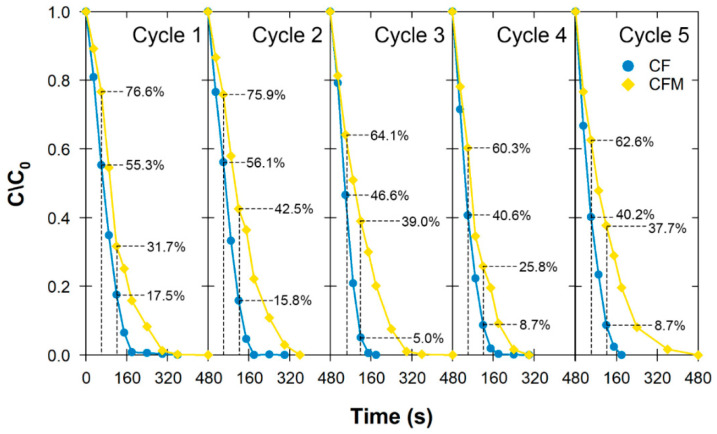
Degradation of BP following five cycles of pumping BP-contaminated water through the catalytic ozonation system using CF or CFM (each catalyst: 0.2 g L^−1^, [BP_o_]: 10 µM, pH 7, 25 °C).

Temporal changes in the BP concentrations showed that BP removal was completed in each cycle in 150–180 s for CF and in 300–360 s for CFM (Figure 7). Notably, the BP degradation in this five-cycle experiment was much faster than in the batch experiment (Figure 5 vs. Figure 7) for two reasons: (1) BP was not the sole contaminant in the batch experiment and (2) the size discrepancy of the contact tank, which may have required a larger O_3_ flux for large-scale application. 

The %removal from the CF-O_3_ system remained the same in cycles 3–5, while that of the CFM-O_3_ system caused fluctuation in the %removal for both selected timelines. Either way, lower catalytic ozonation performance was observed for CFM. A mass-to-mass comparison revealed that CF provided much better performance than CFM even though manually removing CF may result in a slight loss of catalyst compared to using a neodymium magnet to remove CFM from the solution. During ozonation, the CFM aggregation may cause a reduction in reactive sites for O_3_ molecule transfer to generate reactive oxygen species, thereby causing lower degradation performance. Despite that the most beneficial aspect of using CFM was its retractability using a neodymium magnet during the manufacturing process, an additional calcination step was required. Therefore, CF would be more suitable for large-scale production as the additional calcination step is not needed.

### 3.6. Proposed Mechanisms

#### 3.6.1. Enhanced Ozonation Mechanisms

**Figure 8:**
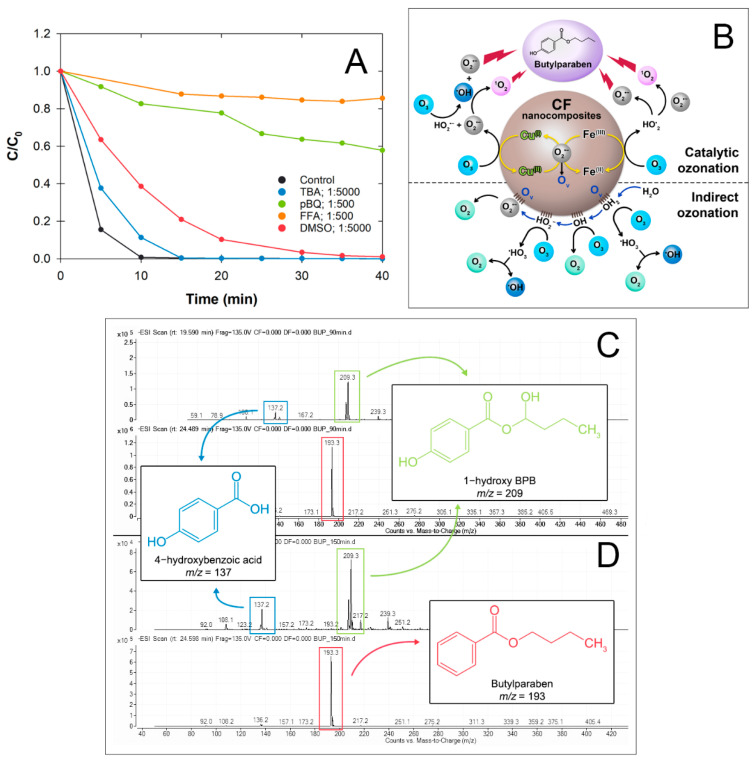
Following catalytic ozonation using O_3_/CF system: (**A**) effects of four scavenging agents on BP removal (catalyst: 0.2 g L^−1^, [BP_o_]: 10 µM, pH 7, 25 °C), and (**B**) proposed catalytic mechanism of O_3_/CF for BP degradation; (**C**,**D**) mass spectra of BP major transformation products from LC–MS at (**C**) 90 min and (**D**) 150 min.

In our experimental setup, two possible catalytic mechanisms emerge: (1) direct/indirect ozonation and (2) heterogeneous catalytic ozonation. To explain the catalytic mechanisms, we used a scavenging experiment to confirm the type of active species using several chemical probes and the XPS spectrum to elucidate the changes in element compositions (Figure 4 and Figure 8A). The results showed that the BP degradation rates were retarded in the following order TBA < DMSO << pBQ < FFA, indicating that BP degradation was mainly from ^1^O_2_ and O_2_^•−^, which corresponded to the lowest BP degradation rates, and ^•^OH was the least predominant radical species. High proportions of oxygen components in the O 1s spectrum of CF-a and CFM-a were observed, indicating strong hydroxylation on the catalyst surface during the ozonation process, which confirmed that these radicals resulted from initiation from the surface hydroxyl groups arising from the indirect ozonation. Electron transfer could also be observed from the Cu 2p and Fe 2p spectra (Cu^(II)^/Cu^(I)^ and Fe^(III)^/Fe^(II)^), which can lead to the abstraction of hydrogen to produce reactive superoxide oxygens (Figure 4) [39]. This coincided with the decrease in O_latt_ (O^2−^) and the increase in O_ads_ caused by the adsorption of O-containing molecules or some small amounts of parabens and their degradates that could be chemically adsorbed before degrading (Figure 4). This also resulted in an increase in oxygen vacancy (O_v_) which later formed –OH_2_ on the surface (CF–OH_2_) (Equation (10)). Then, the O_3_ adsorption and decomposition occurred on the CF surface, causing a release of O_3_ free radicals (^•^HO_3_), conversion of ^•^O^2−^, regeneration of O_v_, and subsequently, the release a new generation of O^2−^ (Equations (11)–(15); [37]) This was a benefit of the existence of several metal oxides in the nanocomposite that had both strong Lewis acid sites and oxygen vacancy sites [40].

O_v_ + H_2_O → CF–OH_2_ (10)

CF–OH_2_ + O_3_ → CF–OH + ^•^HO_3_ (11)

CF–OH + O_3_ → CF–HO_2_^−^ + O_2_ (12)

CF–HO_2_^−^ + O_3_ → CF–^•^O_2_^−^ + ^•^HO_3_ (13)

CF–^•^O_2_^−^ → O_2_ + O_v_ (14)

O_2_ + 4e^−^ → 2O_2_^−^ (15)

According to the above analysis on the regeneration of O^2−^, the electron transfer mechanisms of our ternary nanocomposite during catalytic ozonation are proposed (Figure 8B). Initially, O_3_ adsorption on the surface hydroxyl group occurred on the metal ions (CF≡Cu^(I)^–OH and CF≡Fe^(III)^–OH). Changes in the redox pairs of Fe^(II)^/Fe^(III)^ and Cu^(I)^/Cu^(II)^ were more obvious for CF/CF-a than for CFM/CFM-a, indicating that more electron capture occurred on the CF surface than for CFM and that it may have caused higher catalytic activity for CF in the ozonation process (Figure 4). The loss of an electron from Cu^(I)^ on the CuO could result in the formation of Cu^(II)^ along with the decomposition of O_3_, which later generated both ^•^OH and ^•^O_2_^−^ and later self-reacted to form ^1^O_2_, as the major active species (Figure 8A) (Equations (16)–(19)) [41,42,43]. However, in addition, the ^•^OH was also responsible for this oxidation process (Equation (18)). This was confirmed by the changes in the BP degradation rates (Figure 8A) and the changes in the IR spectra of CF-a at 3400 cm^−1^ (Figure 3A).

CF≡Cu^(I)^–OH + O_3_ → CF≡Cu^(I)^–OH–O_3_ (16)

O^2−^ + CF≡Cu^(I)^–OH–O_3_ → CF≡Cu^(II)^ + HO_2_^•−^ + ^•^O_2_^−^ (17)

O_3_ + HO_2_^•−^ → ^•^OH + ^•^O_2_^−^ + O_2_ (18)

^•^OH + ^•^O_2_^−^ → ^1^O_2_ + OH^−^ (19)

On the other hand, O_3_ interaction with Fe(III) on the Fe_2_O_3_ surface would result in ^•^OH_ads_ and O_3_^•^ (Equation (20)) [44]. Then, electron transfer occurred from Fe(III) to Fe(II), which corresponded to the increase in Fe(II) from the Fe 2p spectrum (Equation (21); Figure 4). Once these O species were formed with the available O_3_ or ^•^OH, they could react and later form both ^1^O_2_ and O_2_^•−^ (Equations (19), (22)–(24)) [17,43].

CF≡Fe^(III)^–OH + O_3_ → CF≡Fe^(III)^–O_3_^•^ + ^•^OH_ads_ (20)

CF≡Fe^(III)^–O_3_^•^ + OH^−^ → CF≡Fe^(II)^ + HO_2_^•^ + O_2_ (21)

HO_2_^•^ → ^•^O_2_^−^ + H^+^ (22)

^•^O_2_^−^ + HO_2_^•^ → ^1^O_2_ + OOH^−^ (23)

HO_2_^•^ + HO_2_^•^ → ^1^O_2_ + H_2_O_2_ (24)

Cu^(II)^ + ^•^O_2_^−^ → Cu^(I)^ + O_2_ (25)

In CFM, the regenerated ^•^O_2_^−^ could, in turn, reduce Cu^(II)^ to Cu^(I)^ via Equation (25); then, there was less O_2_^−^ for the generation of ^1^O_2_ to begin the BP degradation, thus resulting in lower degradation efficiency [41].

#### 3.6.2. Degradates of Paraben Catalytic Ozonation

Aliquot samples of BP following catalyzed-O_3_ treatment at 30, 90, and 150 min were analyzed using LC–MS to determine the degradation products. All initial concentrations were five times higher than the degradation efficiency experiment to warrant the emergence of degradation products. Chromatograms showed that the BP peak (t_R_ 20.12 min) decreased with time and only a trace amount of BP was found at 150 min. The mass spectra of the various BP degradates showed the molecular ion peaks of *m*/*z* 209.3 for 1-hydroxy BPB (t_R_ 24.32 min) (Figure 8C) and *m*/*z* 137.2 for 4-hydroxybenzoic acid (t_R_ 16.73 min). The 1-hydroxy BPB was highest at 90 min of reaction and continued to decrease at 150 min (Figure 8D). 4-Hydroxybenzoic acid was detected at 30, 90, and 150 min. 

**Figure 9:**
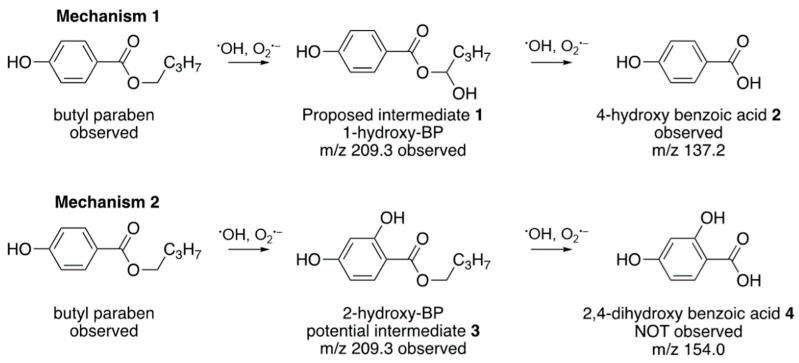
Proposed reaction pathways for the catalytic ozonation of butyl paraben.

There are two likely mechanisms for the degradation of BP based on literature precedent. Previous work [27,39,45] supports Mechanism 1, featuring oxidation by O_3_, ^•^OH, and/or ^•^O_2_^−^, at the alkyl chain to give intermediate 1, and subsequent hydrolysis to the 4-hydroxy benzoic acid 2 (Figure 9). Asgari et al. [46] report that both O_3_ and ^•^OH can give Mechanism 2, where the aromatic ring is first oxidized to give intermediate 3, which could then hydrolyze to give 2,4-dihydroxybenzoic acid 4 (Figure 9). However, Asgari’s work showed continued attack by ^•^OH at the aromatic ring of 4 to give polyhydroxylated degradation products without the hydrolysis of the butyl ester group.

The present data only support Mechanism 1, in that *m*/*z* signals supporting both compound 1 and compound 2 are observed, and are consistent with previous work. While the first step of Mechanism 2 could be supported by assignment of the signal at *m*/*z* 209.3 to potential intermediate 3, this is not supported by other evidence. Compound 4 would be the obvious next product under the same conditions, but is not observed. No other polyhydroxylated compounds are observed. Thus, Mechanism 1 is best supported by the present evidence. 

### 3.7. Toxicological Assessment

**Figure 10:**
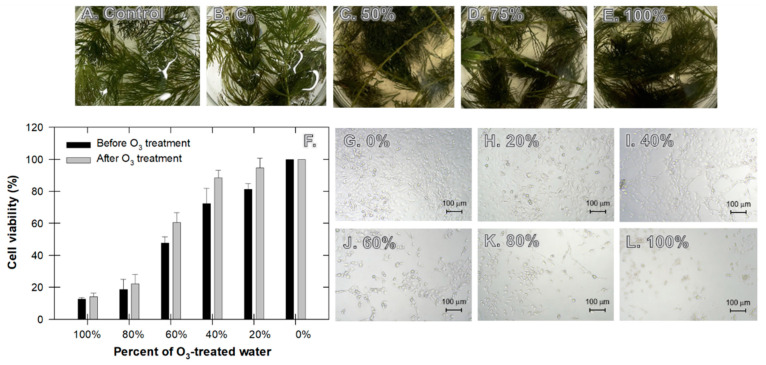
After exposure of samples to different percentages of O_3_-treated water: (**A**–**E**) visual observation of *Ceratophyllum demersum* L., (**F**) cytotoxicity assay of Eker Leiomyoma Tumor-3 (ELT3) cell line, and (**G**–**L**) optical microscopy images of ELT3 cells.

In general, treated wastewater is mixed with natural receiving water in varying amounts dependent upon the water treatment regime. To estimate the toxicological effects of these effluents, we used the changes in physical observation of *Ceratophyllum demersum* L. and the dose-dependent cytotoxicity in ELT3 cells exposed to the various components of O_3_-treated water (0–100%). The control, healthy *Ceratophyllum demersum* L., showed a uniform green color for the entire length of the stem; in contrast, at 5 days of sampling, the older parts of the plants were slightly detached, and the young tip was more resistant with 50% of treated water than with 100% of treated water (Figure 10A–E), perhaps because the tested water did not significantly harm the plants, supporting catalytic O_3_ as a clean treatment technology. The detachment of *Ceratophyllum demersum* L. leaves is the primary indicator of its death following exposure to toxic chemicals, but this incidence usually occurs after long-term exposure (>14 d) or a sudden high dose of chemicals. Chen et al. [47] observed negative changes when the plants were submerged in 40–80 mM of lead solution after 21 days. In our case, O_3_ and its reactive species have a very short half-life; therefore, the direct effect on the plants was minimal.

The question remaining is whether this O_3_-treated water is safe for other living organisms that are far more sensitive to *Ceratophyllum demersum* L. In an attempt to answer this question, we used ELT3 cells as representative cells. No samples exerted general cytotoxicity (<80% cell viability) at 0–20% of O_3_-treated water (Figure 10F). In addition, we found that the cytotoxicity potential of the O_3_-treated water was higher than for the untreated water by approximately 15% (Figure 10F), indicating a negative consequence from the O_3_ treatment. The viability discrepancy was more pronounced for a smaller component of O_3_-treated water, which corresponded to the attachment of cells seen using microscopic evaluation (Figure 10G–L). At 0–40% of the O_3_-treated mixture, the attachment of cells was clear, representing their better viability. This observation corresponded to the viability percentage, which implied that by decreasing O_3_-treated water from 60% to 20%, the viability reached 96%. Given that the bioassay results showed that at >40% O_3_-treated water, effluent samples can be toxic to tested cells, while, to a lesser extent, cell deterioration was unlikely. 

Although parabens have been reported to cause cytotoxic effects on human peripheral lymphocytes, it was likely to occur within 1–2 days of exposure [48]. Cells were reported to undertake mitochondrial dysfunction following exposure to various concentrations of parabens [49]. Under our experimental conditions, we believe the 4-hydroxybenzoic acid and 4-hydroxybenzoic decreased viability of ELT3 cells in a dose-dependence manner. Given that the parent parabens were rapidly removed, the cause of algal morphological alteration and an increase in cell mortality were from the paraben degradates, with the toxicological effects being reduced by these three important factors: (1) a larger dose of O_3_ flux, (2) a longer contact time, and (3) a smaller proportion of treated water. A sequential release of treated water is suggested, and the cell deterioration mechanisms should be thoroughly investigated in the future study.

## 4. Conclusions

In the present study, the ternary CuFe_2_O_4_/CuO/Fe_2_O_3_ nanocomposites (CF), synthesized using co-precipitation methods, were used for the catalytic ozonation process to treat paraben-contaminated water. Constructing graphitic carbon nitride (g-C_3_N_4_) in the composites by the calcination of melamine (CFM) during synthesis resulted in improved material magnetism. The catalytic ozonation efficiency and recyclability performance of both materials were revealed. The O_3_/CF system could effectively remove all parabens from contaminated water within minutes, while CF displayed better performance for degrading parabens than CFM; however, the CFM had better reusability after five cycles. The presence of organic and inorganic constituents had both positive and negative effects on butylparaben degradation rates due to the presence of secondary active radicals. The main dominant reactive oxygen species in the system were ^1^O_2_ and ^•^O_2_^−^, which are initiated from the ^•^OH generated through indirect ozonation. Through the scavenging experiment and changes in element composition from XPS, the catalytic ozonation of CuFe_2_O_4_/CuO/Fe_2_O_3_ nanocomposite catalytic ozonation was also proposed. The O_3_/CF system has proven that it is a clean technology and can be applied to conventional water treatment, as it shows no effects on both *Ceratophyllum demersum* L. and Eker Leiomyoma Tumor-3 cells at up to 40% composition with clean water. For larger-scale treatment in a real-world application, the O_3_:CF system is recommended, because it provides a promising catalytic ozonation process without the need for a large O_3_ supply and without toxic by-products.

## Data Availability

The authors confirm that the data supporting the findings of this study are available within the article.

## Supplementary Materials

The following supporting information can be downloaded at: https://www.mdpi.com/article/10.3390/nano12203573/s1, Section S1. Chemical vendors and manufacturers; Section S2. Analytical procedures; Section S3. Catalyst characteristics; Section S4. Preparation of ELT3 cell culture; Figure S1. Degradation of methylparaben (MP) under different treatment systems; Figure S2. Degradation of ethylparaben (EP) under different treatment systems; Figure S3. Degradation of propylparaben (PP) under different treatment systems; Figure S4. Particle size distribution of: (A) CF, (B) CFM, and (C) N_2_ adsorption/desorption isotherms of both catalysts; Figure S5. Survey scan of XPS spectra of the CuFe_2_O_4_/CuO/Fe_2_O_3_ ternary nanocomposites before and after catalytic ozonation; Figure S6. Temporal changes in parabens at varying CF dosages in the O_3_/CF system Tables: Table S1. Basic water quality of real-word swimming pool water; Table S2. Average particle size obtained from XRD analysis; Table S3. Average particle size obtained from particle size distribution using TEM analysis.
