# Supplementary material for: Fabrication of Ternary Nanoparticles for Catalytic Ozonation to Treat Parabens: Mechanisms, Efficiency, and Effects on Ceratophyllum demersum L. and Eker Leiomyoma Tumor-3 Cells
Source: Nanomaterials (Basel). 2022 Oct 12;12(20):3573. doi: 10.3390/nano12203573 (PMC9610848; doi:10.3390/nano12203573)
Supplement: Supplementary file 1 [file nanomaterials-12-03573-s001.zip › nanomaterials-1909789-supplementary.pdf]

nanomaterials

Supplementary Materials

Fabrication of Ternary Nanoparticles for Catalytic Ozonation to Treat Parabens: Mechanisms, Efficiency, and Effects on *Ceratophyllum demersum* L. and Eker Leiomyoma Tumor-3 Cells

Apiladda Pattanateeradetch [1], Chainarong Sakulthaew [2], Athaphon Angkaew [1], Samak Sutjarit [2], Thapanee Poompoung [2], Yao-Tung Lin [3], Clifford E. Harris [4], Steve Comfort [5] and Chanat Chokejaroenrat [1,*]

1 Department of Environmental Technology and Management, Faculty of Environment, Kasetsart University, Bangkok 10900, Thailand
2 Department of Veterinary Technology, Faculty of Veterinary Technology, Kasetsart University, Bangkok 10900, Thailand
3 Department of Soil & Environmental Sciences, National Chung Hsing University, Taichung, Taiwan
4 Department of Chemistry and Biochemistry, Albion College, Albion, MI 49224, USA
5 School of Natural Resources, University of Nebraska-Lincoln, Lincoln, NE 68583, USA
* Correspondence: chanat.c@ku.ac.th; Tel.: +66-2579-3877

S1. Chemicals Vendors and Manufacturers

Four parabens were used (purity ≥ 99%) as target contaminants (methylparaben; MP, ethyl 4-hydroxybenzoate; EP, propyl 4-hydroxybenzoate; PP, and butyl 4-hydroxybenzoate; BP). MP, PP, and BP were purchased from Sigma-Aldrich (Burlington, MA, USA). Melamine ($C_3H_6N_6$), EP, and P-benzoquinone (pBQ) were obtained from Alfa Aesar (New Taipei, Taiwan). Copper sulfate ($CuSO_4$), zinc sulfate ($ZnSO_4{\bullet}7H_2O$), and calcium chloride ($CaCl_2$) were purchased from Ajax Finechem (Auckland, New Zealand). Ferric chloride hexahydrate ($FeCl_3{\bullet}6H_2O$) was purchased from QRëC (Auckland, New Zealand). Sodium hydroxide (NaOH) was purchased from Ecfmsure (Darmstadt, Germany). Tert-butanol (TBA), Sodium bicarbonate ($NaHCO_3$), and sodium chloride (NaCl) were obtained from Carlo Erba (Val-de-Reuil, France). Furfuryl alcohol (FFA) was purchased from Acros Organics (Geel, Belgium). Dimethyl sulfoxide (DMSO) was purchased from RCI Labscan (Bangkok, Thailand). Trichloroisocyanuric acid (TCCA) was obtained from a local vendor in Bangkok, Thailand. Ammonium acetate ($NH_4CH_3CO_2$) and manganese sulfate monohydrate ($MnSO_4{\bullet}H_2O$) were purchased from Loba Chemie (Mumbai, India). Cobalt (II) chloride hexahydrate ($CoCl_2{\bullet}6H_2O$) was purchased from Asia Pacific Specialty Chemicals Ltd (Botany, New South Wales, Australia). Acetonitrile (ACN; HPLC grade) was purchased from Fisher Scientific (Seoul, Korea). Ethanol (EtOH) and methanol (MeOH; reagent grade, 99.9%) and nickel (II) chloride hexahydrate ($NiCl_2{\bullet}6H_2O$) were purchased from Daejung (Gyeonggi, Korea). Distilled water (DI) was used to prepare the aqueous solution and HPLC mobile phase.

S2. Analytical Procedures

Temporal changes in parabens were analyzed using high-performance liquid chromatography (HPLC) in conjunction with a UV photodiode array detector (e2695, 2998, Waters, Milford, MA, USA). The injection volume of samples was set at 20 µL and the mobile phase ratio was 60:40 (ACN:DI). Peak separation was achieved with isocratic elution at a flow rate of 1 mL/min using a C18 column (Hypersil ODS, 250 mm × 4.6 mm, 5 µm). Paraben detection occurred at 254 nm, with retention times of 3.6, 4.1, 4.6, and 5.5

min for methylparaben (MP), ethylparaben (EP), propylparaben (PP), and butylparaben (BP), with detection limits of 14.86, 8.11, 8.80, and 18.97 μg L^{-1}, respectively.

The O_3-treated samples were concentrated prior to LC–MS analysis. Each ProElut C18 SPE cartridge (Dikma, Foothill Ranch, CA, USA) was placed on a vacuum manifold (Agela, Torrance, CA, USA) and pre-conditioned with MeOH (5 mL) and milli-Q water (5 mL). Then, 30 mL of each sample was loaded through a cartridge at a constant flow rate of 1 mL/min. The concentrated components were eluted twice with MeOH (2 mL) at the same flow rate. Then, the extract was transferred to an LC vial using a 0.45 μm PTFE syringe filter. Chromatographic separation of degradates was performed using a triple quadrupole LC–MS (Agilent 6420; Santa Clara, CA, USA) with the same column as used in the HPLC analysis. The mobile phase was 0.1% ammonium acetate in MeOH: DI water (80:20). The total flow rate was set at 0.2 mL min^{-1}. Mass spectral data were obtained on an LC quadrupole mass spectrometer with an electrospray ionization (ESI) source performed in negative ion mode.

S3. Catalyst Characteristics

The morphological properties of the nanocomposites were investigated using a scanning electron microscope (SEM; JOEL, JSM-6010, Tokyo, Japan) with energy dispersive X-ray spectroscopy (EDS) and transmission electron microscopy (TEM; Thermo Scientific, TALOS F200X, Waltham, MA, USA); subsequently, the corresponding particle size distributions were determined. The crystal structures of nanomaterials were characterized using a D2 phaser Bruker X-ray diffractor (Bruker, Berlin, Germany). The surface functional groups were determined using Fourier- transform infrared spectroscopy (FTIR; Tensor 27, Bruker, Berlin, Germany) and Raman spectroscopy (FT-Raman, Bruker, RAM II, Billerica, MA, USA) while chemical compositions were investigated using X-ray photoelectron spectroscopy (XPS; Kratos, AXIS ultra DLD, Manchester, England). A vibrating sample magnetometer was specifically customized by the Department of Physics, Kasetsart University, Bangkok, Thailand, and used for material magnetic characterization. Two types of ternary nanocomposites (CF and CFM) before and after catalyzation in the O_3 system were selectively characterized using these instruments.

S4. Preparation of ELT3 Cell Culture

The basal medium used in growing ELT3 cell cultures is a 1:1 mixture of Dulbecco's modified Eagle's medium (DMEM) and Nutrient Mixture F-12 (HAM) (DMEM/F12). A complete medium volume of 500 mL contained 56 mL of Gibco fetal bovine serum (FBS) from Thermo Fisher Scientific (Waltham, MA, USA) with a final concentration of 1.6 μM ferrous sulfate, 50 nM sodium selenite, 12 nM vasopressin, 10 nM cholesterol, 0.2 μM hydrocortisone, 10 μg mL^{-1} transferrin, 1 nM 3,3′,5-triiodo-L-thyronine sodium salt (T3), and 25 μg mL^{-1} insulin. Most of the ingredients were purchased from Sigma-Aldrich (St. Louis, MO, USA). Cells were cultured under humidified atmosphere with 5% CO_2 in an incubator at 37 °C.

To determine the effects of O_3-treated water on ELT3 cell viability and growth, $1x10^4$ cells were seeded in a 96-well plate per well and incubated for 24 h. These cells were then exposed to a different mixture of treated water varying from 0 to 100% for 24 h in 5% CO_2 incubator at 37 °C. We used MTS cell proliferation assay kit (ab1917010, Abcam, UK) to detect cell viability. Once the culture period was reached, the old medium was discarded and replaced with new medium. Then, 20 μL MTS reagent was added into each well and incubated for 1 to 4 h at 37 °C in the dark. The absorbance at 450 nm was measured using microplate reader (Bio-Rad, Hercules, CA, USA). Comparison of the optical density of each sample was evaluated. Cell viabilities were graphically demonstrated along with the cell morphological property using an inverted microscope (Olympus, CKX53, Shinjuku, Tokyo, Japan).

Table S1. Basic water quality of real-world swimming pool water.

Real waters	pH	SAL (psu)	TDS (g/L)	Conductivity (mS/cm)
Chlorine pool water	7.78	0.3	0.31	0.58
Saline pool water	7.90	2.7	3.10	5.31
Natural receiving water	7.74	0.3	0.35	0.68
Municipal wastewater	7.76	0.4	0.50	0.94

Table S2. Average particle size obtained from XRD analysis.

Catalyst	Average Particle Size (nm)
CF	23.43
CF-a	24.26
CFM	21.17
CFM-a	21.05

Table S3. Average particle size obtained from particle size distribution obtained from TEM analysis.

Catalyst	Mean (nm)	Min (nm)	Max (nm)	Median (nm)
CF	25.2	10.72	41.81	25.38
CFM	20.84	10.249	71.159	18.118

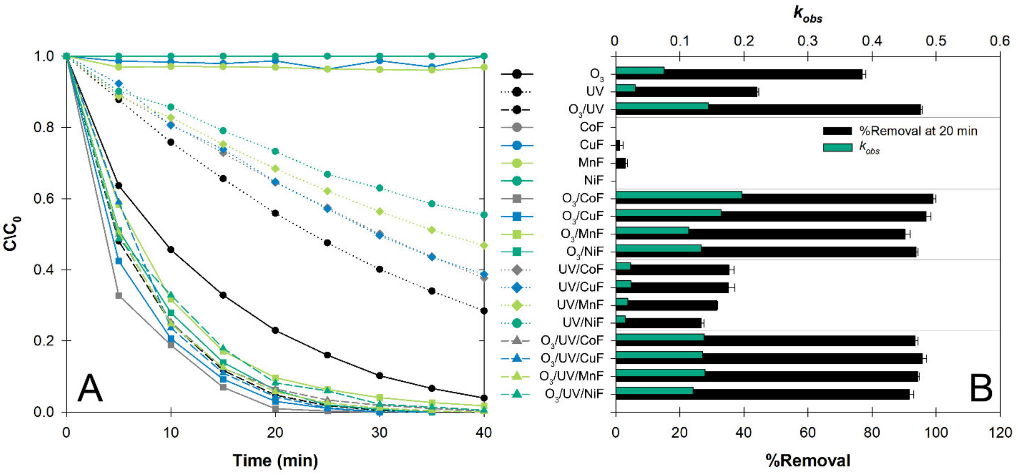

Figure S1. Degradation of methylparaben (MP) under different treatment systems: (**A**) degradation kinetics, and (**B**) observed rate constant (k_{obs}) and %removal at 20 min (catalyst: 0.2 g L^{-1}, [MP_0]: 10 μM, pH 7, 25 °C). Error bars indicate ± standard deviation.

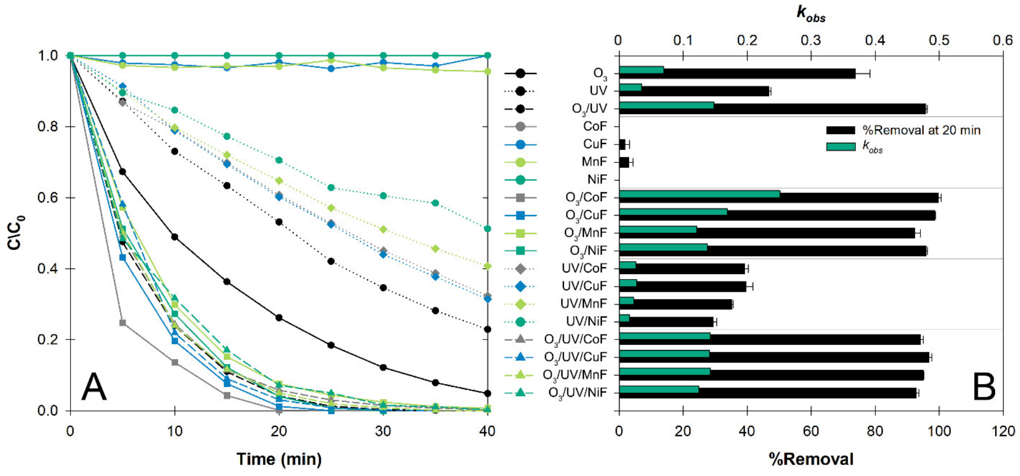

Figure S2. Degradation of ethylparaben (EP) under different treatment systems: (**A**) degradation kinetics, and (**B**) observed rate constant (k_{obs}) and %removal at 20 min (catalyst: 0.2 g L^{-1}, $[EP_o]$: 10 μM, pH 7, 25 °C). Error bars indicate ± standard deviation.

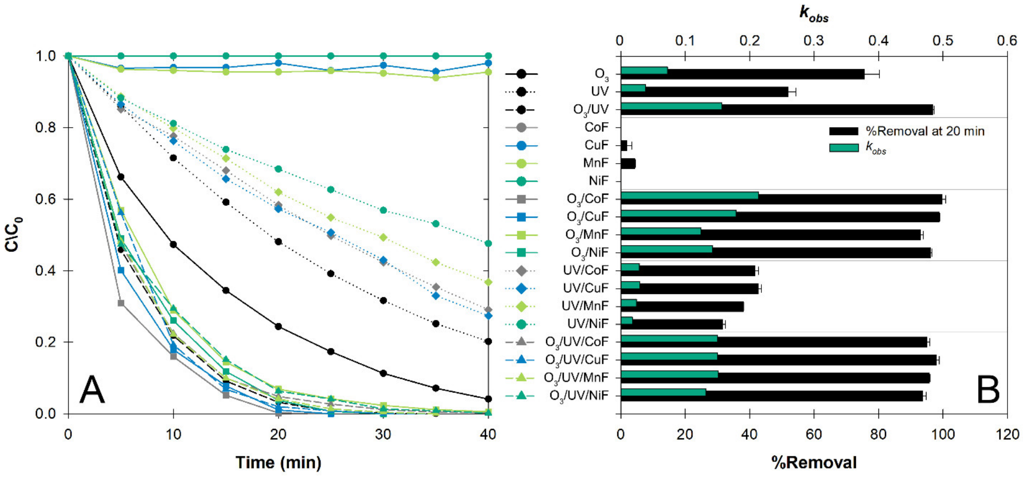

Figure S3. Degradation of propylparaben (PP) under different treatment systems: (**A**) degradation kinetics, and (**B**) observed rate constant (k_{obs}) and %removal at 20 min (catalyst: 0.2 g L^{-1}, $[PP_o]$: 10 μM, pH 7, 25 °C). Error bars indicate ± standard deviation.

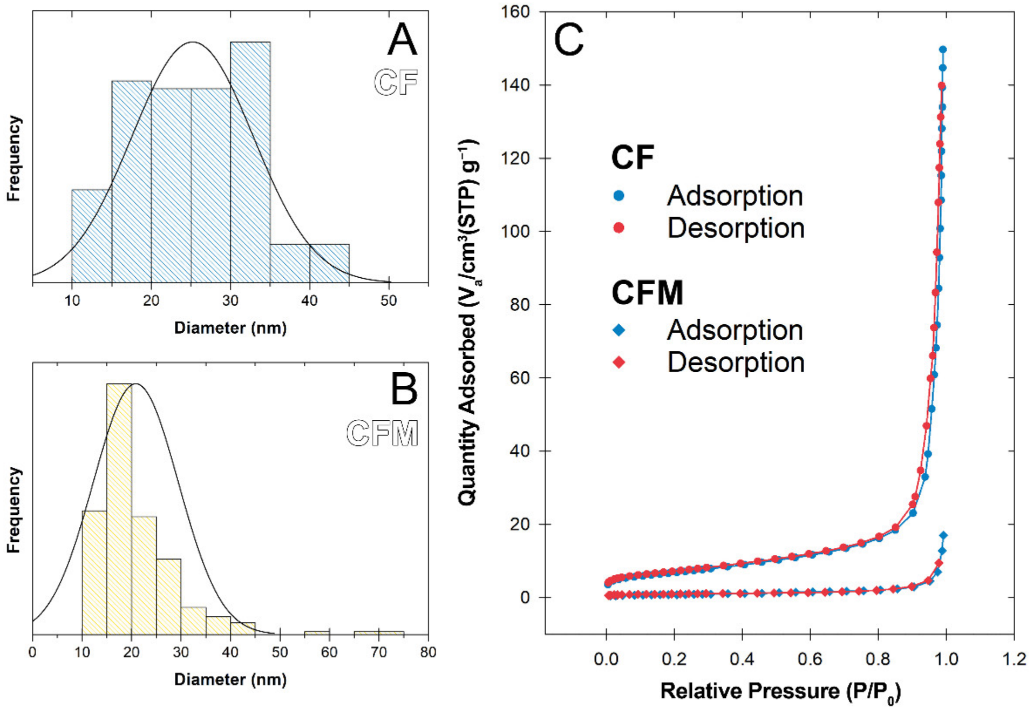

Figure S4. Particle size distribution of: (**A**) CF, (**B**) CFM, and (**C**) N_2 adsorption/desorption isotherms of both catalysts.

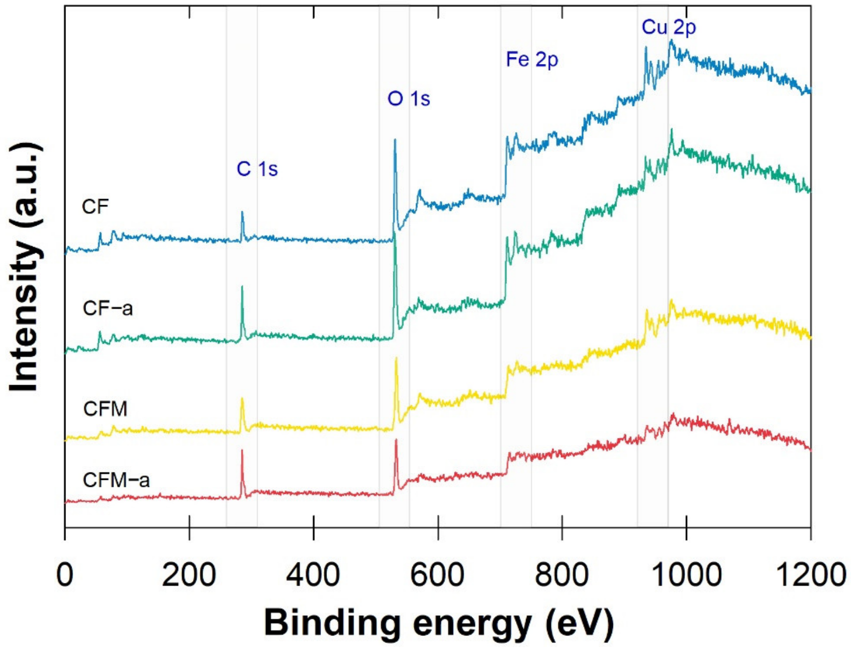

Figure S5. Survey scan of XPS spectra of the $CuFe_2O_4/CuO/Fe_2O_3$ ternary nanocomposites before and after catalytic ozonation.

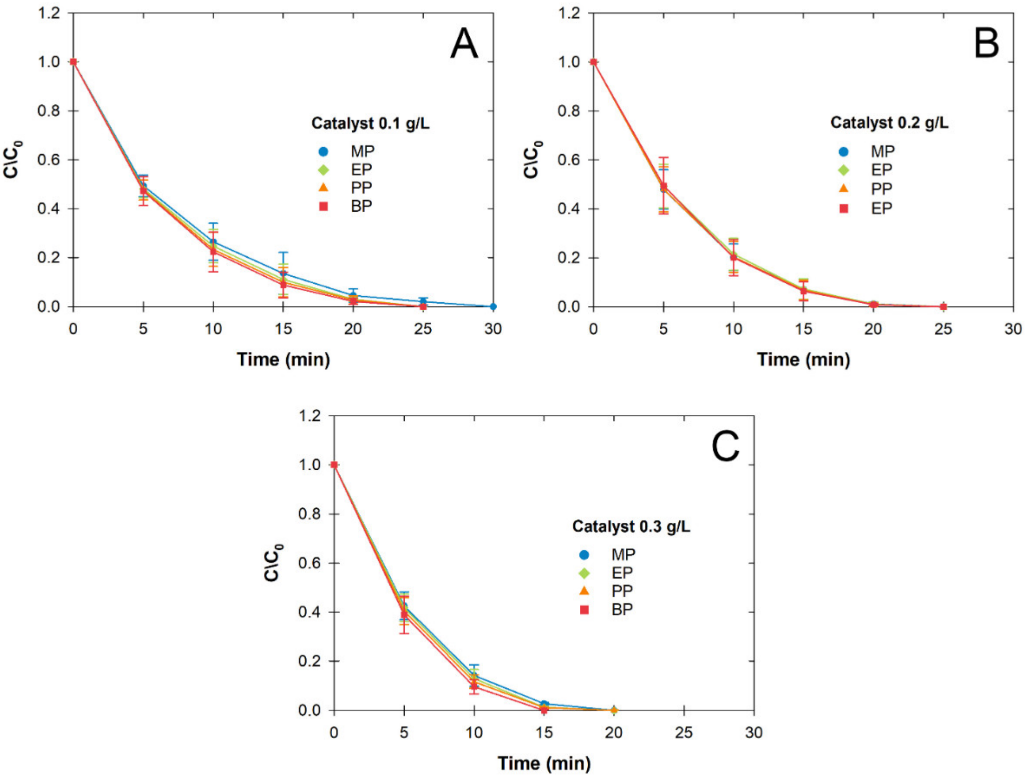

Figure S6. Temporal changes in parabens at varying CF dosages in the O_3/CF system: (**A**) 0.1 g L^{-1}, (**B**) 0.2 g L^{-1}, and (**C**) 0.3 g L^{-1} ([each $paraben_o$]: 10 μM, pH 7, 25 °C). Error bars indicate ± standard deviation.
